# Major Anthropogenic Causes for and Outcomes of Wild Animal Presentation to a Wildlife Clinic in East Tennessee, USA, 2000–2011

**DOI:** 10.1371/journal.pone.0093517

**Published:** 2014-03-31

**Authors:** Ashley N. Schenk, Marcy J. Souza

**Affiliations:** Department of Biomedical and Diagnostic Sciences, University of Tennessee College of Veterinary Medicine, Knoxville, Tennessee, United States of America; University of Florida, United States of America

## Abstract

To determine the reasons for presentation and outcome of wildlife cases in East Tennessee, a retrospective analysis was performed using 14,303 records from cases presented to the wildlife clinic of the University of Tennessee Veterinary Teaching Hospital between 2000 and 2011. The cases were first categorized into amphibian/non-avian reptile, mammal, or avian and then classified into groups based on the primary admitting/presenting sign. There are a variety of reasons animals were presented to the clinic, and some were directly or indirectly anthropogenic in origin, including cat related, dog related, hit by automobile, and other human encounters leading to trauma; of the cases reviewed, 4,443 (31.1%) presented for one of these 4 reasons. Overall case fatality risk in regard to these 4 admitting/presenting signs was 0.519 for the amphibian/non-avian reptile cases, 0.675 for mammal cases, and 0.687 for avian cases. This study confirms the importance of monitoring wildlife morbidity and mortality and of focusing efforts to reduce the anthropogenic threat on native habitats and resident wildlife populations.

## Introduction

Around the world, wildlife species and the ecosystems they inhabit are evolving and shifting in an attempt to adapt to human influences and environmental change. The factors dictating these changes are diverse and numerous, making it difficult to separate the effect each has on species survivorship. Increased morbidity and mortality of wildlife can be attributed to a variety of factors, and many of these are related to human activities such as land development and usage, predation by domestic pets, and automobile traffic [1]–[4].

As the urbanization of areas with native wildlife continues to increase, numerous pressures are exerted on the natural structures of those habitats. For example, infringing development may not only increase exposure between wildlife species, domestic animals, and invasive species, but may also increase the exposure of wild animals to novel pathogens, leading to what Daszak et al. call “pathogen pollution” [1]. Clearing and isolating habitats through the development of high-traffic roadways can increase roadside automobile-related mortalities, noise, environmental pollution, and disturbance stress on the surrounding wildlife [5]. The construction of other infrastructure can change temperature variations as well as the flow of water runoff thereby increasing flooding and further deteriorating the habitable environment [6]–[8]. The establishment of neighborhoods and housing communities will likely also increase the number of domestic animals in the area, which provides an unnatural predator stress and possibly novel infectious agents on nearby native species.

Regardless of whether these increasing threats to wildlife originate directly or indirectly from anthropogenic effects, or as a result of disease spread, it is important to understand the extent of their impact. One study found that trauma and infection were the main reasons eastern box turtles were presented to a wildlife clinic [9]. Similar studies were conducted to investigate reasons wild raptors and reptiles were admitted to wildlife rehabilitation centers, and both studies found evidence of anthropogenic origins of trauma [10], [11]. The purpose of this study was to investigate the reasons wildlife were presented to a veterinary medical center to determine the greatest anthropogenic causes of morbidity and mortality.

## Methods

There were 14,943 records reviewed from wildlife cases that presented to the University of Tennessee Veterinary Teaching Hospital between January 2000 and November 2011. 640 cases were omitted; these included cases in which “Dead on Arrival” or “Euthanasia” were the only details given for the reason for presentation as well as rechecks and cases in which there was insufficient data for categorization. The 14,303 records remaining included species or species group (“songbird” being the most detailed animal group indicated on many avian records), and these species or species groups were classified according to type of animal (amphibian/non-avian reptile, mammal, or avian). The cases in which cat related, dog related, hit by automobile, or human-induced traumatic incidents were mentioned in the primary admitting/presenting sign (A/PS) were separated out and analyzed. These signs were derived from information provided by the person admitting the animal and do not represent a final diagnoses made by the clinician, but they do provide detailed information about the patient’s condition and the most likely origin of injury.

The A/PSs were grouped as follows: Human-induced trauma cases included those which mentioned gunshot, fence entrapment, fishing line or hook injury, lawn mower or weed eater encounter, and trapped as a non-target species cases. Cat related, dog related, and automobile related cases were separated out as well in order to show their frequency. Any time “hit by car” or “found in road” was indicated, the animal was placed in the hit by automobile category. Cat and dog related cases were categorized accordingly and included explanations such as “attacked by dog”, “found in cats mouth”, and “killed by cat.” The number of cases in each category was determined, and the outcomes (alive, dead on arrival [DOA], died, or euthanized) were recorded. Animals classified as “alive” were either released directly by hospital personnel or transferred to a rehabilitation facility; only cases determined to have a good or excellent prognosis for release were transferred to a rehabilitation facility. The placement of non-releasable animals from the veterinary teaching hospital into education facilities is extremely rare.

## Results

Of the 14,303 cases evaluated, 4,333 (31.1%) were classified as presenting for one of the 4 major A/PSs evaluated; case frequencies ranged from 12 to 1,115 within the animal groups, and the case fatality risk ranged from 0.316 to 0.836 (Table 1). Overall case fatality risk in regards to these 4 focus A/PSs was 0.519 for the amphibian/non-avian reptile cases, 0.675 for mammal cases, and 0.687 for avian cases. Hit by automobile cases had the highest fatality risk (0.715), followed by cat-related injury cases (0.675), human-induced trauma cases (0.603), and dog-related cases (0.600) across all animal groups. Although cat-related cases had the highest percent of natural deaths following presentation to the clinic, hit by automobile cases had the highest percent of cases with successive euthanasia (Table 1).

**Table 1:** Case outcomes and case fatality risks (CFR) for human-induced trauma, cat related, dog related, and hit by automobile cases for amphibian/non-avian reptile, mammal, and avian animals presented to a wildlife clinic in East Tennessee.

	n (% of cases)		ALIVE (%)		DOA (%)		DIED (%)		EUTHAN (%)		CFR
AMPHIBIAN/NON-AVIAN REPTILE	397		192		3		32		171		0.519
1. Human-Induced Trauma	60	(15.1)	41	(68.3)	0	(0.0)	3	(5.0)	16	(26.7)	0.316
2. Cat Related	12	(1.5)	8	(66.7)	0	(0.0)	1	(8.3)	3	(25.0)	0.333
3. Dog Related	50	(6.1)	31	(62.0)	0	(0.0)	3	(6.0)	17	(32.0)	0.380
4. Hit by Automobile	275	(33.3)	112	(40.7)	3	(1.1)	25	(9.1)	135	(49.1)	0.593
**MAMMAL**	2318		754		36		263		1265		0.675
1. Human-Induced Trauma	111	(4.8)	32	(28.8)	2	(1.8)	13	(11.7)	64	(57.7)	0.712
2. Cat Related	1115	(19.4)	388	(34.8)	9	(0.8)	166	(14.9)	552	(49.5)	0.652
3. Dog Related	597	(10.4)	253	(42.4)	13	(2.2)	52	(8.7)	279	(46.7)	0.576
4. Hit by Automobile	495	(8.6)	81	(16.4)	12	(2.4)	32	(6.5)	370	(74.7)	0.836
**AVIAN**	1738		544		23		299		872		0.687
1. Human-Induced Trauma	202	(11.6)	75	(37.1)	0	(0.0)	27	(13.4)	100	(49.5)	0.629
2. Cat Related	809	(10.5)	232	(28.7)	9	(1.1)	168	(20.8)	400	(49.4)	0.713
3. Dog Related	244	(3.2)	73	(29.9)	3	(1.2)	39	(16.0)	129	(52.9)	0.701
4. Hit by Automobile	483	(6.3)	164	(34.0)	11	(2.3)	65	(13.5)	243	(50.3)	0.660

## Discussion

Wildlife species are continually being presented to veterinary clinics and rehabilitation centers throughout the United States, and it is important to determine the reasons in order to monitor the changing health status of the surrounding ecosystem [10], decrease the anthropogenic effect of habitat fragmentation and pathogen pollution ([2], [12]–[14], and investigate preemptive strategies for reducing the number of wildlife casualties. This large dataset provides a sample to explore causal trends for presentation and sheds light on some of the major anthropogenic threats to wildlife health. This study does not attempt to explain the origin or cause of all reasons for presentation, but rather focuses on human related causes of presentation.

Approximately 1/3 of the cases examined were presented to the hospital because of either direct or indirect anthropogenic reasons. Direct interactions with humans (human induced trauma and hit by automobile categories) were less common than indirect interactions (dog and cat categories) in this population, but still made up 11% of the total cases. Pathogen pollution, noise pollution, and environmental pollution have also been shown to lead to wildlife morbidity and mortality [1], [15]–[17], but this study provides an additional explanation that “predator pollution,” by means of introducing domestic cats and dogs to wildlife areas, may also be having a profound and damaging effect. Of all cases presented, approximately 20% were due to interactions with domestic pets, specifically cats (14% of all cases) and dogs (6% of all cases). By narrowing the interface between wild and urbanized areas, it is likely that human-wild animal encounters, whether direct or indirect, will increase, and based on the results of this study, these encounters frequently result in the detriment of the wild animals.

The data provided in this study does not investigate or provide evidence for the role of environmental pollution, pesticide use, or other forms of habitat disruption, but it does lend itself to the needed discussion about the many factors contributing to the morbidity and mortality of native wildlife species. In order to establish long-term conservation, a variety of initiatives including responsible pet ownership and habitat modification should be considered.

Community and veterinary-client education about the importance, as it relates to wildlife, of keeping domestic cats indoors and preventing domestic dogs from roaming outside unsupervised could lead to a reduction in the number of animals presented to wildlife facilities based on the findings of this study [18]. Although pets other than dogs and cats were not identified as reasons for presentation in this study, exotic, invasive species can lead to wildlife morbidity and mortality in other regions. Providing educational materials to owners about the proper care of their exotic pets may decrease those introduced to the wild by intentional abandonment and therefore reduce interactions with native wildlife [19].

Increasing canopy coverage and the shrub layer along urban parks and greenways has been suggested to increase crucial habitat areas for certain avian species and protect them from the negative pressures of urbanized areas [20]. In addition, evidence supports certain habitat defragmentation projects, such as linear patches and biological corridors, as successful in increasing migratory ranges and establishing connectivity between wildlife [5], [21], [22]. On a smaller scale, establishing larger wildlife-friendly areas by arranging neighborhood gardens adjacent to each other has also been proposed as a means to increase wildlife habitat in urbanized areas [23]. By removing invasive predators, focusing efforts on the conservation of native habitats, and affording a level of protection along developed and undeveloped transition zones, the numbers of animals affected by direct and indirect interactions with humans might be decreased, therefore leading to decreased morbidity and mortality.

## Conclusion

This study examined the causes for wildlife submission to a wildlife clinic to understand the patterns and trends of human related reasons for presentation. Through this and other studies, it is apparent that anthropogenic factors, including land development, as well as direct interactions with humans, automobiles, and invasive predators are important causes of wildlife morbidity and mortality [3], [4], [12], [14]. Because final diagnosis was assumed from the A/PS in our study and because some signs lacked explanatory detail, additional studies reviewing comprehensive patient records, as well as detailed clinician diagnoses, may provide stronger evidence supporting patterns for wildlife presentation to veterinary clinics and rehabilitation centers. It is also important to understand that many of these cases were submitted by “Good Samaritans” and this may present a bias in the case data. The animals brought to the clinic were likely found in easily accessible, populated areas, and this may lead to a misrepresentation of the causes of morbidity and mortality in less-developed areas. In addition to defragmenting habitats and establishing biological corridors, it is important to remove invasive species and contain domestic animals in order to decrease the predatory stress they impose. Through agendas like these and a more mindful approach to land development planning, the anthropogenic threat to wildlife species might be minimized.

## References

[1] Daszak P, Cunningham AA, Hyatt AD (2001) Anthropogenic environmental change and the emergence of infectious diseases in wildlife. Acta Trop 78: 103–116. 11230820 10.1016/s0001-706x(00)00179-0

[2] Pavlacky DC Jr, Possingham HP, Lowe AJ, Prentis PJ, Green DJ, et al.. (2012) Anthropogenic landscape change promotes asymmetric dispersal and limits regional patch occupancy in a spatially structured bird population. J Anim Ecol DOI:10.1111/j.1365-2656.2012.01975.x. 10.1111/j.1365-2656.2012.01975.x 22489927

[3] Robertson I (1998) Survey of predation by domestic cats. Aus Vet J 76: 551–554. 10.1111/j.1751-0813.1998.tb10214.x 9741724

[4] Ament R, Clevenger A, Yu O, Hardy A (2008) An assessment of road impacts on wildlife populations in U.S. national parks. Environ Manage 42: 480–496. 18437455 10.1007/s00267-008-9112-8

[5] Trocmé M (2006) Habitat fragmentation due to linear transportation infrastructure: An overview of mitigation measures in Switzerland. Proceedings of the 6th Swiss Transport Research Conference. Monte Verita/Ascona, Switzerland, pp 1–20.

[6] Barron OV, Barr AD, Donn MJ (2013) Evolution of nutrient export under urban development in areas affected by shallow watertable. Sci Total Environ. 443: 491–504 10.1016/j.scitotenv.2012.10.085 23220139

[7] Taylor J, Lai KM, Davies M, Clifton D, Ridley I, et al (2011) Flood management: prediction of microbial contamination in large-scale floods in urban environments. Environ Int 37(5): 1019–1029 10.1016/j.envint.2011.03.015 21481472

[8] Lumpkin HA, Pearson SM (2013) Effects of exurban development and temperature on bird species in the southern Appalachians. Conserv Biol 27: 1069–1078 10.1111/cobi.12085 23773053

[9] Schrader GM, Allender MC, Odoi A (2010) Diagnosis, treatment, and outcome of eastern box turtles (*Terrapene carolina carolina*) presented to a wildlife clinic in Tennessee, USA, 1995–2007. J Wildl Dis 46: 1079–1085. 20966259 10.7589/0090-3558-46.4.1079

[10] Molina-López RA, Casal J, Darwich L (2011) Causes of morbidity in wild raptor populations admitted at a wildlife rehabilitation centre in Spain from 1995-2007: a long term retrospective study. PLoS One 6: e24603. 21966362 10.1371/journal.pone.0024603 PMC3179465

[11] Brown JD, Sleeman JM (2002) Morbidity and mortality of reptiles admitted to the Wildlife Center of Virginia, 1991 to 2000. J Wildl Dis 38: 699–705. 12528435 10.7589/0090-3558-38.4.699

[12] Leu M, Hanser SE, Knick ST (2008) The human footprint in the west: A large-scale analysis of anthropogenic impacts. Ecol Appl 18: 1119–1139. 18686576 10.1890/07-0480.1

[13] Segelbacher G, Manel S, Tomiuk J (2008) Temporal and spatial analyses disclose consequences of habitat fragmentation on the genetic diversity in capercaillie (*Tetrao urogallus*). Mol Ecol 17: 2356–2367. 18422931 10.1111/j.1365-294X.2008.03767.x

[14] Daszak P, Cunningham AA, Hyatt AD (2000) Emerging infectious diseases of wildlife–Threats to biodiversity and human health. Science 287: 443–449. 10642539 10.1126/science.287.5452.443

[15] Tsipoura N, Burger J, Newhouse M, Jeitner C, Gochfeld, et al (2011) Lead, mercury, cadmium, chromium, and arsenic levels in eggs, feathers, and tissues of Canada geese in the New Jersey Meadowlands. Environ Res 111: 775–784. 21679937 10.1016/j.envres.2011.05.013

[16] Arlettaz R, Patthey P, Baltic M, Leu T, Schaub M, et al (2007) Spreading free-riding snow sports represent a novel serious threat for wildlife. Proc R Soc Lond B Biol Sci 274: 1219–1224. 10.1098/rspb.2006.0434 PMC2189568 17341459

[17] Romano MC, Rodas AZ, Valdez RA, Hernández SE, Galindo F, et al (2010) Stress in wildlife species: Noninvasive monitoring of glucocorticoids. Neuroimmunomodulation 17: 209–212. 20134205 10.1159/000258726

[18] Cooper CB, Dickinson J, Phillips T, Bonney R (2007) Citizen Science as a tool for conservation in residential ecosystems. Ecol Soc 12: 11.

[19] Jenkins PT (1996) Free Trade and Exotic Species Introductions. Conserv Biol 10: 300–302.

[20] U.S. Fish and Wildlife Service (2003) Recovery Plan for the Red-cockaded Woodpecker (*Picoides borealis*): Second Revision. Atlanta, GA: U.S. Fish and Wildlife Service.

[21] Shepard B, Whittington J (2006) Response of wolves to corridor restoration and human use management. Ecol Soc 11:1. Available: http://www.ecologyandsociety.org/vol11/iss2/art1/. Accessed July 27, 2012.

[22] Rosenberg DK, Noon BR, Meslow EC (1997) Biological corridors: Form, function, and efficacy. BioScience 47: 677–687.

[23] Goddard MA, Dougill AJ, Benton TG (2010) Scaling up from gardens: biodiversity conservation in urban environments. Trends Ecol Evoln 25: 90–98. 10.1016/j.tree.2009.07.016 19758724

